# Nanostructure Design of Catalysts: Latest Advances and Prospects

**DOI:** 10.3390/nano13131980

**Published:** 2023-06-30

**Authors:** Zuzeng Qin

**Affiliations:** School of Chemistry and Chemical Engineering, Guangxi University, Nanning 530004, China; qinzuzeng@gxu.edu.cn

The recent development of nanotechnology has laid the foundation for the design and preparation of various nanostructured materials. The use of nanostructured materials has surpassed that of classical bulk-structured materials due to their unique chemical, physical, electrical, and mechanical properties and their excellent tunability. The nanostructural design of catalysts is the most popular topic at the forefront of catalysis research due to the unique characteristics of nanocatalysts, such as their surface effect, volume effect, and quantum size effect, and their catalytic activity and selectivity are much better than those of conventional catalysts [1,2,3]. In the fields of thermocatalysis, electrocatalysis, and photocatalysis, nanomaterial-based catalysts have attracted much attention due to their relatively high efficiency and stability. The nanostructural design of catalysts focuses on composition regulation, size optimization, morphology control, structural engineering, interface engineering, etc., as well as the excellent catalytic performance brought about by nanoscale catalysts [4]. At present, the control and design of specific properties, i.e., morphology, size, porosity, electrical conductivity, optical properties, photoelectric properties, chemical activity, etc., of nanometer materials have encountered many challenges. Therefore, this Special Issue will introduce the latest progress and prospects of catalyst nanostructure design, aiming to gain a deeper understanding of the catalyst structure–activity relationship to better solve the problems arising in practical applications.

This Special Issue of Nanomaterials, entitled “Nanostructure Design of Catalysts: Latest Advances and Prospects”, combines relevant research and review contributions that cover the latest nanocatalysts. This Special Issue covers thermal catalysis [5,6,7,8], photocatalysis [9], electrocatalysis [10], and adsorbent nanomaterials [11,12,13]. It contains six research papers on nanometer materials for catalysts, including for thermal catalysis, photocatalysis, electrocatalysis, and other fields, which provide some new ideas for the nanostructure design of catalysts. At the same time, it also contains three research papers on nano-adsorbent materials, whose unique three-dimensional mesh structures have large specific surface areas and could be used as carriers of active components of catalysts, meaning they have great application value.

Water pollution is a major problem that urgently needs to be solved in modern society. Advanced oxidation processes (AOPs) are considered to be one of the most promising methods to remove organic pollutants from the environment. For this purpose, Angel-Vasile et al. [5] carbonized polyaniline (PANI) at 900 °C to prepare thermally treated polyaniline (PANI 900) and compared the presence of polyaniline at different pH values (4, 7, and 10) for the catalytic ozonation of an ibuprofen solution. The catalyst was characterized comprehensively by a variety of techniques (SEM, Raman spectroscopy, XPS, pHPZC, etc.), while the oxidation process of the ibuprofen solution (100 mg L^−1^) was evaluated by several analytical methods (HPLC, UV254, TOC, COD, and BOD5). The results showed that the removal rate of ibuprofen was significantly increased in the presence of PANI 900 (20 min at pH 10) compared with the noncatalytic process (56 min at pH 10). Moreover, the ozone depletion of mgO_3_/mg ibuprofen during the catalytic process (17.55-PANI, 11.18-PANI 900) was significantly lower than that without a catalyst (29.64). The pH value of the solution had a certain effect on the degradation process, indicating that the removal rate of organic substrates is higher in the alkaline region than in the acidic or neutral range.

In addition to water pollution, the Earth is rich in biomass resources, among which the decomposition of agricultural waste in the soil not only causes certain environmental pollution, but also wastes resources. To solve this problem, Elena et al. [6] prepared nanocomposites based on 13X zeolite (13XZ), calcium oxide (CaO), and metallic zinc particles (Zn). Catalytic and noncatalytic pyrolysis experiments on residual rapeseed biomass (RRB) were conducted, and the effects of nanocomposites on the yield and properties of liquid–solid products were established. The results show that the surface area (S_BET_) of the nanocomposite was reduced by introducing CaO and metal zinc particles into the 13X zeolite, and this reduction was due to the introduction of nano-calcium carbonate and nano-zinc particles, which occupy an important space in the zeolite structure. The elemental analysis showed that the main elements in the nanocomposite were Si, Al, Ca, and Zn, which proves that the molecular sieve structure was preserved. These types of nanocomposites can be used as catalysts in the pyrolysis process and could be used in the pyrolysis process of biomass to obtain higher quality bio-oil and to ensure good characteristics and stability.

In a different study, to solve the problem of organic reactions in water, Vladimir et al. [7] proposed a novel method for the synthesis of NHC PEPPSI metal surfactants based on the functionalization of imidazole 4,5-dicarboxylic acid, hydrophilic oligoethylene glycol, and lipophilic alkyl fragment sequences. The results showed that the most lipophilic complex in the reduction reaction was three times more active than the commercial PEPPSI complex, which was related to the formation of monodisperse aggregates detected by DLS and TEM methods.

The surface of nNi is easily oxidized during the preparation of nano-nickel powder by the liquid-phase reduction method. For this reason, Bao et al. [8] improved the preparation of nano-nickel (nNi) powder by using anhydrous ethanol as the solvent and hydrazine hydrate as the reducing agent. In addition, HTPB/nickel composites were prepared using hydroxy-terminated polybutadiene (HTPB) as a coating agent. The introduction of HTPB will not change the crystalline form of nickel. After coating, the active nickel content of Ni nanoparticles increased from 45.0% to 69.0%, and the application of Ni nanoparticles was greatly improved. The catalytic behavior of HTPB/Ni for the thermal decomposition of ammonium perchlorate (AP) was investigated by differential scanning calorimetry (DSC) and thermogravimetric analysis (TGA). The results showed that HTPB/Ni has little effect on the thermal decomposition of AP at low temperatures, but the peak value of thermal decomposition at high temperatures increased from 456 °C to 332 °C.

In addition to the above research on thermal catalysis, photocatalysis technology has attracted wide attention due to its environmental friendliness. Lara et al. [9] modulated the photocatalytic activity of surface ligands by using three carboxylic acids (tartaric acid, benzoic acid, and citric acid) in the sonochemical synthesis process to achieve control over their size and morphology. The results showed that α-Ag_2_WO_4_ synthesized with citric acid has a nanoscale size and can degrade 100% RhB within 60 min at a concentration of 2 mg/mL. Removal experiments showed that hydroxyl radicals •OH and cavitation ℎ^+^ were the main biological oxidants. The recovery test showed that the α-Ag_2_WO_4_-CA nanomaterial was stable after four cycles.

Similarly, due to the continual development of sustainable clean energy technology, electrocatalysis technology has great application prospects. Saravanan et al. [10] prepared graphene by pyrolysis using graphene oxide (GO), and the oxygen reduction reaction (ORR) activity in a 0.1 M KOH aqueous solution was superior to the electrocatalytic activity of the original GO. Various graphene samples were prepared by pyrolysis of 50 mg and 100 mg GO in three alumina boats at 900 °C in a N_2_ atmosphere. The prepared samples were named from G50-1B to -3B and G100-1B to G100-2B. The results showed that G100-1B and -2B had an excellent electrocatalytic performance compared with GO and G50-1B to -3B. Compared with Pt/C electrodes (E_onset_, E_1/2_, J_L_, and n values of 0.965, 0.864, 5.222, and 3.71), G100-1B (E_onset_, E_1/2_, J_L_, and n values of 0.843, 0.774, 4.558, and 3.76) and G100-2B (E_onset_, E_1/2_, J_L_, and n values of 0.837, 0.737, 4.544, and 3.41) showed better electrocatalytic ORR activity. The findings point to the potential use of graphene as an electrocatalytic oxygen reduction reaction in fuel cells and metal–air batteries.

Different from the above catalysts, the following nanocomposites mainly use natural polymers as raw materials, and the corresponding three-dimensional network structures were designed through the polymerization reaction to not only have a huge specific surface area but also have excellent surface chemical properties while realizing the reuse of waste. Regarding the modification of water-absorbing nanomaterials, Xie et al. [11] used a silane coupling agent to modify nano-CaCO_3_ (MNC) and N,N′-methylene bisacrylamide (MBA) as a double crosslinking agent. By grafting acrylamide/acrylic acid (AM/AA) onto bagasse cellulose, an AM/AA composite super-absorbent agent (CAAMC) was prepared. CAAMC has a 3D network structure, and its maximum water absorption rate is 712 g/g in deionized water and 72 g/g in 0.9% NaCl solution. The FT-IR and XPS results showed that nano-CaCO_3_ was modified by a silane coupling agent and MNC was chemically bonded to inorganic components. By adjusting the content of MNC, the structure of CAAMC can be changed, and the mechanical properties, reusability, and water retention of CAAMC can be improved. Adding a small amount of CAAMC to the soil can effectively improve the water retention capacity of the soil, indicating that CAAMC has great application potential in soil water retention.

In another work by Xie et al. [13], magnetic tapioca-starch porous microspheres (AAM-MSMPMs) were prepared by graft copolymerization in the inverse emulsion. The AAM-MSMPM adsorbent is superparamagnetic with a saturation magnetization of 14.9 emu·g^−1^, and can be quickly and directionally separated from Cd(II) ions in an aqueous solution. FTIR showed that carboxyl and hydroxyl groups were successfully grafted onto MS and that AAM-MSMPM had good permeability and a uniform size. After the addition of porogen, the particle size of AAM-MSMPM decreased from 19.00 nm to 7.00 nm, the specific surface area increased from 7.00 to 35.00 m^2^·g^−1^, and the pore volume increased from 0.03 to 0.13 cm^3^·g^−1^. AAM-MSMPM has a large specific surface area and provides more adsorption active sites for Cd(II) ions. The maximum adsorption capacity of AAM-MSMPM for Cd(II) ions was 210.68 mg·g^−1^, which was 81.02% higher than that without a pore-causing agent. This study showed that AAM-MSMPM is a promising adsorption material.

Finally, the study by Xie et al. [12] used (3-chloro-2-hydroxypropyl) trimethyl ammonium chloride as a cationic reagent. POCl_3_ was used as an anionic reagent, which was prepared by the W/O microemulsion crosslinking method of amphoplastic cassava starch nanoparticles (CA-CANPs). The anionic and cationic groups were successfully introduced into CA-CSNPs by FTIR, NMR, and P element detection. Via zeta-potential testing, it was also determined that CA-CSNPs have a charge reversal function and pH response, which can be controlled by adjusting the molar ratio of nitrogen to phosphorus (pl). In addition, the loading and release properties of CA-CSNPs with pl 6.89 were evaluated by using PTX as a model drug. PTX achieved a loading rate of 36.14 mg·g^−1^ and a release rate of 37.61% within 96 h in 0.9% normal saline at a pH = 7.0. This means that CA-CSNPs can be used as a biocompatible and biodegradable controlled release vector of antitumor drugs and have the potential to carry out targeted drug control on tumor cells.

Overall, the nine papers included in this Special Issue on nanostructure catalysts discuss the use of nanotechnology to improve catalyst performance and introduce their application prospects.
